# The Emergency Medicine Education and Research by Global Experts (EMERGE) Network: Challenges and Lessons Learned

**DOI:** 10.5811/westjem.2022.7.56398

**Published:** 2022-10-18

**Authors:** Prashant Mahajan, Shu-Ling Chong, EMERGE Network, Vijaya Arun Kumar, Prerna Batra, Apoorva Belle, Ben Bloom, Chung-Hsien Chaou, Ulf Ekelund, Sagar Galwankar, Johanna Kaartinen, Vimal Krishnan, Qingbian Ma, Paul M. Middleton, Anna Miethke Morais, Chip Jin Ng, Daniel Osei-Kwame, Dominik Roth, Rasha Sawaya, Sanjeev Singh, Tej Prakash Sinha, Mabel Vasnaik, Katie Walker, Adriana Yock

**Affiliations:** * University of Michigan, Department of Emergency Medicine, Ann Arbor, Michigan; † KK Women’s and Children’s Hospital, Department of Emergency Medicine, Singapore; ‡ University of Michigan, Emergency Medicine Education and Research by Global Experts (EMERGE) Network, Ann Arbor, Michigan; Wayne State University; UCMS & GTP Hospita; University of Michigan; Royal London Hospital; Chang Gung Memorial Hospital; Skane University at Lund, Lund University; Florida State University; University of Helsinki and Helsinki University Hospital; Kasturba Medical College; Peking University Third Hospital; South Western Emergency Research Institute; Sao Paulo University; Chang Gung Memorial Hospital; Komfo Anokye Teaching Hospital; University of Vienna; American University of Beirut Medical Center; South Western Emergency Research Institute; Sao Paulo University; Chang Gung Memorial Hospital; Komfo Anokye Teaching Hospital; University of Vienna

## Abstract

**Introduction:**

The Emergency Medicine Education and Research by Global Experts (EMERGE) network was formed to generate and translate evidence to improve global emergency care. We share the challenges faced and lessons learned in establishing a global research network.

**Methods:**

We describe the challenges encountered when EMERGE proposed the development of a global emergency department (ED) visit registry. The proposed registry was to be a six-month, retrospective, deidentified, minimal dataset of routinely collected variables, such as patient demographics, diagnosis, and disposition.

**Results:**

Obtaining reliable, accurate, and pertinent data from participating EDs is challenging in a global context. Barriers experienced ranged from variable taxonomies, need for language translation, varying site processes for curation and transfer of deidentified data, navigating institution- and country-specific data protection regulations, and substantial variation in each participating institution’s research infrastructure including training in research-related activities. We have overcome many of these challenges by creating detailed data-sharing agreements with bilateral regulatory oversight agreements between EMERGE and participating EDs, developing relationships with and training health informaticians at each site to ensure secure transfer of deidentified data, and formalizing an electronic transfer process ensuring data privacy.

**Conclusion:**

We believe that networks like EMERGE are integral to providing the necessary platforms for education, training, and research collaborations for emergency care. We identified substantial challenges in data sharing and variation in local sites’ research infrastructure and propose potential approaches to address these challenges.

## INTRODUCTION

Research in emergency medicine (EM) has increased in complexity and sophistication through collaborative efforts in the past two decades.^1^ Specifically, research networks can provide adequate cohort size for statistical power, global representation of illnesses for generalizability, and structured research support to ensure integrity and quality of study designs.^2^ Research networks can overcome current barriers such as varying research infrastructure support in emergency departments (ED) across the globe and allow for evidence generation.^3^

The Emergency Medicine Education and Research by Global Experts (EMERGE) network was formed in June 2018, with the goals of generating evidence, translating knowledge to improve emergency care for patient populations, and strengthening EM research capacity globally.^4^ In the spirit of being inclusive we have reduced barriers to entry; thus, all institutions with EDs regardless of their annual census or academic affiliations can become EMERGE members. EMERGE continues to attract member sites across all six World Health Organization regions and continents, with a current membership of 26 EDs in 17 countries (www.EMERGENetwork.org). Aggregating high quality data on a common platform regardless of geographic borders is critical for describing and comparing the basic epidemiology of emergency care globally; increasing numbers to power hypothesis-generating studies; and improving generalizability of research findings.^5^ Here we report the issues and barriers faced by the network and discuss potential solutions.

## METHODS

Operating on a carousel model,^6^ EMERGE is governed by an executive committee consisting of three subcommittees aligned with the missions of research, education, and data oversight (performed by a data coordinating center). EMERGE intentionally proposed the development of a global ED visit registry to demonstrate feasibility of data-sharing. The registry is a six-month, retrospective, deidentified, minimal dataset of routinely collected variables, such as patient demographics, diagnosis, and disposition. The data was collected by determining the core elements a priori, and each site communicated with the data-coordinating center to harmonize variable fields from their respective electronic health records (EHR).

## RESULTS

EMERGE experienced considerable barriers ranging from variable data taxonomies, need for language translation, varying processes for data cleaning and transfer of deidentified data, and navigating numerous data protection regulations (Table 1). Institutions and countries vary substantially regarding data oversight, ranging from the need to set up individualized research agreements to legal barriers resulting in the inability to transfer deidentified data across geographic borders for some participating sites. Such data-fencing has precluded some sites from participating in the EMERGE registry.

We have overcome many of these challenges through creating detailed data-sharing agreements with bilateral regulatory oversight, developing relationships with and training site health informaticians to ensure secure transfer of deidentified data, and formalizing a transfer process ensuring data privacy. Currently, 18/19 EMERGE sites in 13 countries have institutional review board approval, 13/18 have provided initial sample data while 8/13 have provided complete six-month data (Supplement Table 1). In most instances, after meeting site regulatory requirements for data transfer, sites reported difficulties in extracting data from their EHRs. The EMERGE data coordinating center had to take on roles beyond data curation and analyses and is now working with each individual institution’s information technology teams across EMERGE sites to ascertain data quality and authenticity. We also found substantial variation in each participating institution’s research infrastructure including training in research-related activities (eg, good clinical practices, study design and statistical analyses, grant writing). and resources including statisticians and research associates.

## DISCUSSION

EMERGE encountered substantial challenges in obtaining high quality data across its participating sites in a timely manner, which is an inherent barrier toward generating evidence and improving emergency care globally. Barriers encountered ranged from restrictive regulatory data governance to lack of time and support for emergency clinicians to participate. Despite the barriers we identified in this article, EMERGE was able to quickly respond and conducted a pandemic preparedness study among 26 member and 103 non-member sites. By leveraging the EMERGE network, many more sites were recruited via referrals and direct solicitation, and all participants who were approached agreed to participate in the study.^7^ This supports the notion that EDs across the globe want to participate in endeavors to generate evidence.

Because it is an unfunded network, such intense data efforts from EMERGE will require substantial resources and are unsustainable. We believe the future viability of international research networks will depend on developing a federated data model in which the data is collected using standardized definitions and processes, retained at the institution, analysed locally or using federated machine learning, and reported in an aggregated manner while preserving privacy and overcoming regulatory requirements.^8^ However, based on our experience, building a federated data model requires sites to obtain appropriate local regulatory approvals and have the necessary data infrastructure including data scientists and trained personnel to support this approach.

Another approach to enhancing site research capabilities is to enhance the research training and education of the personnel in each participating ED. We are currently collaborating with the Development Implementation, and Assessment of Novel Training in Domain-based competencies (DIAMOND), which is a web-based, curated research education platform, developed by the Clinical & Translational Awards (CTSA) mechanism in the United States.^9^ This novel and scalable platform allows research personnel to evaluate their knowledge gaps and build highly customizable, on-demand, web-based research education modules for training in research methods and procedures. The enhanced research methodology training will increase the site support and thereby sustain engagement in global research participation. Sharing of data-related resources across sites will further enhance the success of the federated data model.

## LIMITATIONS

The barriers encountered and solutions proposed are based on our experience, and it is possible that research networks in other specialities or those that have more robust support may have different barriers and challenges. Our experience is that with limited resources, there is a risk of over-burdening the sites as well as the central data-coordinating center. Some of these barriers can be potentially circumvented by site commitment of time and resources to the network research goals and aligning priorities.^10^. EMERGE has collected information from participating sites through an ED demographics study that will allow us to better delineate each site’s patient population and research capabilities to participate in studies and thereby facilitate decisions on the type and number of active studies at any one point in time for the network.^11^

## CONCLUSION

We identified challenges in data-sharing and variation in research infrastructure among sites. Immediate next steps include the need to create regulatory-compliant federated data models, enhance research education and training, develop relevant research priorities, and identify research questions that require global participation yet can be performed at sites with limited resources.

## Supplementary Information



## Figures and Tables

**Table 1:** Issues faced in data collection across a global research network and potential solutions.

	Issues	Potential Solutions
Global level	Political unrest Infections (eg, pandemics) Different languages Need for international funding	Remain sensitive to the impact of politics on research personnel, infrastructure, and timelines Adopt an opportunistic research posture Provide translation services Conduct needs analysis and seek appropriate funding channels
National level	Data regulations (eg, GDPR^a^, ICMR^b^, LGDP^c^) Regulatory compliance – Ethics Committee	Build a federated data model Provide guidance using a master study protocol and guidance documents
Regional level	Application of national laws Data variables differ	Understand the variability in regional interpretation of national laws Accept data variables in variable formats; provide data consultation services
Site level	Variability in requirements by ethics committees Data governance and concerns of breach in confidentiality Trust issues	Provide research education via DIAMOND platform Work on site-specific protocol templates; understand the concerns and differing requirements of various ethics boards Communication with specific sites prior to data transfer to eliminate the possibility of receiving identifiable data; create a process system with data center Maintain transparency, provide regular updates
System level	Variability in fields for electronic health records Information technology support/availability	Ensure data compatibility for major variables Consider funding where possible for personnel for data extraction Remain flexible to adapt documents and data use agreements to reflect site-specific requirements that do not diverge from overall data policy
Personnel level	Accessing the Collaborative Institutional Training Initiative Research experience Lack of dedicated administrative and research time	Building a mentor-mentee model Provide authorship and acknowledgements as an incentive

*GDPR*, General Data Protection Regulation, EU data law; *ICMR*, Indian Council of Medical Research, Indian data law; *LGDP*, Lei Geral de Proteção de Dados or General Data Protection Law, Brazil data law; *DIAMOND*, Development, Implementation, and Assessment of Novel Training in domain-based competencies (https://diamondportal.org/).
