# Supplementary material for: The Emergency Medicine Education and Research by Global Experts (EMERGE) Network: Challenges and Lessons Learned
Source: West J Emerg Med. 2022 Oct 18;23(6):947–51. doi: 10.5811/westjem.2022.7.56398 (PMC9683780; doi:10.5811/westjem.2022.7.56398)
Supplement: Supplementary file 1 [file wjem-23-947-s001.docx]

Supplement Table 1: EMERGE member sites and their participation in Emergency Department (ED) visit registry study

| **Country** | **Site** | **Ethics Committee Status** | **Sample Data** | **Full Data** | **Public/Private** |
| --- | --- | --- | --- | --- | --- |
| Australia | Liverpool Hospital | Approved | In Progress | In Progress | Government/Public |
| Austria | Medical University of Vienna | Approved | Yes | In Progress | Government/Public |
| China | Peking University Health Science Center | Approved | Yes | In progress | Government/Public |
| Finland | HUS Helsinki University Hospital | Approved | Yes | Yes | Government/Public |
| Ghana | Komfo Anokye Teaching Hospital (KATH) | Approved | Yes | Yes | Government/Public |
| Hong Kong | The University of Hong Kong | Approved | In Progress | In Progress | Government/Public |
| India | All India Insititue of Medical Sciences (AIIMS) - Delhi | Approved | Yes | In Progress | Government/Public |
| India | Amrita Institute of Medical Sciences | Approved | In Progress | In Progress | Private |
| India | Guru Tegh Bahadur Hospital | Approved | Yes | In Progress | Government/Public |
| India | Kasturba Medical College Manipal | In Process | In Progress | In Progress | Private |
| India | Manipal Hospital Private Limited | Approved | Yes | In Progress | Private |
| Lebanon | American University of Beirut Medical Center | Approved | Yes | Yes | Private |
| Singapore | SingHealth DukeNUS Academic Medical Center | Approved | Yes | Yes | Government/Public |
| Sweden | Skane University Hospital | Approved | In Progress | In Progress | Government/Public |
| Taiwan | Chang Gung Memorial Hospital | Approved | Yes | Yes | Private |
| U.K. | The Royal London Hospital | In Process | In Progress | In progress | Government/Public |
| U.S.A. | Florida State University | Approved | Yes | Yes | Government/Public |
| U.S.A. | University of Michigan | Approved | Yes | Yes | Government/Public |
| U.S.A. | Wayne State University/ Detroit Receiving Center | Approved | Yes | Yes | Government/Public |
